# Sound masking by a low-pitch speech-shaped noise improves a social robot’s talk in noisy environments

**DOI:** 10.3389/frobt.2023.1205209

**Published:** 2024-01-09

**Authors:** Hamed Pourfannan, Hamed Mahzoon, Yuichiro Yoshikawa, Hiroshi Ishiguro

**Affiliations:** ^1^ Intelligent Robotics Laboratory (Hiroshi Ishiguro’s Laboratory), Department of Systems Innovation, Graduate School of Engineering Science, Osaka University, Osaka, Japan; ^2^ Institute for Open and Transdisciplinary Research Initiatives (OTRI), Osaka University, Osaka, Japan

**Keywords:** acoustic satisfaction, human-robot interaction, low-frequency dominance, noisy social spaces, sound masking, speech-shaped noise, user experience

## Abstract

**Introduction:** There has been a surge in the use of social robots for providing information, persuasion, and entertainment in noisy public spaces in recent years. Considering the well-documented negative effect of noise on human cognition, masking sounds have been introduced. Masking sounds work, in principle, by making the intrusive background speeches less intelligible, and hence, less distracting. However, this reduced distraction comes with the cost of increasing annoyance and reduced cognitive performance in the users of masking sounds.

**Methods:** In a previous study, it was shown that reducing the fundamental frequency of the speech-shaped noise as a masking sound significantly contributes to its being less annoying and more efficient. In this study, the effectiveness of the proposed masking sound was tested on the performance of subjects listening to a lecture given by a social robot in a noisy cocktail party environment.

**Results:** The results indicate that the presence of the masking sound significantly increased speech comprehension, perceived understandability, acoustic satisfaction, and sound privacy of the individuals listening to the robot in an adverse listening condition.

**Discussion:** To the knowledge of the authors, no previous work has investigated the application of sound masking technology in human-robot interaction designs. The future directions of this trend are discussed.

## 1 Introduction

In recent years, the use of social robots has increased significantly in public and social spaces (Wada and Shibata, 2006; Korn et al., 2018; Mubin et al., 2018). Social robots can be described as embodied artificial agents that are empowered with artificial intelligence algorithms in the case of autonomous robots, operated by a human agent in the case of teleoperated robots, or are a combination of both in the case of semi-autonomous robots. Some famous examples of such robots include Pepper, NAO, and Robovie and more robots are being developed around the world (Ahmad et al., 2017). These robots are designed to interact with humans and provide guidance, information, and entertainment among other activities. Numerous research initiatives have been undertaken to explore the applications of social robots in public spaces. For instance, they have been deployed as receptionists in hotels to assist guests and answer their inquiries (Zalama et al., 2014), served as tour guides in museums to provide descriptions of artifacts (Faber et al., 2009), and have played diverse roles in healthcare centers to support patients (Wada and Shibata, 2006; Morgan et al., 2022). Tokyo Olympics 2020 was one of the recent examples where robots oversaw guiding passengers during the events as well as in the airport to assist the staff in their endeavours (Srivastava, 2017).

However, one of many challenges of implementing social robots in noisy public spaces is the difficulty in ensuring clear communication between the robot and human in such environments. This is due to the large body of evidence in support of the negative effect of noise on speech comprehension, memory, and learning (Smith, 1985; Jafari et al., 2019) as well as more subjective aspects of the interaction such as developing a negative impression about the speaking person (Kjellberg, 1990). The presence of background noise could negatively affect the comprehension of the presented speech both in the robot and in the human client. This study, however, concentrates on the comprehension of the auditory information presented by the robot to the human user. The negative effect of background noise on the performance of subjects has been observed when using different types of noises including white noise (Bell et al., 2013), vocal and instrumental music (Lehmann and Seufert, 2017), as well as babble noise (Brännström et al., 2018). Still, the most intrusive type of background noise is suggested to be irrelevant background speech (Salamé and Baddeley, 1982). Humans have a special sensitivity towards signals that might carry speech information and this makes ignoring background noise that includes speech signals more difficult than other types of environmental noise (Tremblay et al., 2000).

To reduce the distraction caused by background noises in noisy environments, sound masking technology has been invented (Haapakangas et al., 2011). Sound masking is a process of adding a steady state nonfluctuating background sound to an environment to mask or cover up unwanted noises (Chanaud, 2007). The core idea behind using masking sounds is to make the tempo-spectral properties of the background speech less easily detectable and hence, reduce the distraction caused by them (Mackenzie et al., 2021). A stationary noise with a slope of −5 dB per octave is the most commonly used type of masking sound (Hodgson and Warnock, 1992). Recent trends in sound masking technology have shifted towards using Speech-Shaped Noise (SSN). Speech-shaped noise is a type of stationary noise that follows the spectrum of human speech and hence, results in a better masking power when the background noise includes speech signal (Renz et al., 2018a). SSN is created by applying the Long-Term Average Spectrum of speech on a randomly generated auditory noise.

Sound masking technology has been used for years in open-plan office spaces to improve privacy and reduce distractions (Bradley, 2003). In addition to open-plan offices, sound masking has been implemented in highly populated public spaces such as food courts (Lee and Lee, 2020). This study suggests that people felt more relaxed and described their experience more pleasantly when there was a water fountain sound added to the environmental noises. The same effect to a lesser extent was observed for using the conventional masking sound in the same environment. Furthermore, another study shows that when a masking sound is added to the office environment, subjects report significantly better concentration and cognitive performance in comparison with no added masking sound (Mueller et al., 2021). Other studies have replicated the positive effect of different types of masking sounds on both the indices of the cognitive performance in the subjects such as their speech transmission index, memory error rate, and working memory capacity, as well as their subjective evaluations such as perceived annoyance rate and the perceived sound privacy (Hongisto et al., 2004; Schlittmeier and Liebl, 2015; Renz et al., 2018b).

Despite this relative abundance of evidence in support of the positive effect of adding masking sounds in noisy environments to compensate for the negative effect of background noise in human-human interaction scenarios, the application of masking sounds for improving the functionality of social robots in noisy environments is a new concept. In noisy social spaces, sound masking can enhance communication between the robot and human by reducing the impact of background noise on the speech comprehension of humans about the robot’s speech. This is important because it allows the robot to be heard and understood by humans more readily, which in turn can enhance the overall user experience. This consequently can enhance the perceived reliability of the service robot which makes clients more prone to use such robots again in the future (Desai et al., 2012).

One of the primary benefits of using sound masking technology for social robots is that it enables clear communication in environments where background noise is a common occurrence. For instance, in airports, there is a significant amount of background noise from announcements, people talking, and machinery. This can make it difficult for a social robot to interact with a passenger, especially if the robot is designed to provide time-sensitive information. FRAnny is an example of such a robot that has been recently implemented in Frankfort airport as an initial trial and provides general information such as flight departure times and the nearest accessible restroom or restaurant to passengers in several languages (Furhat, 2022). This robot is equipped with a visual display in front of the robot that augments the presentation of the target information to the person by demonstrating the trajectory that the person should take to reach their requested destination or the time of their intended flight. The number of similar robots in charge of providing requested information to passengers, patients, and customers is only expected to rise in the future.

However, the limitation of current masking sounds is the overall low acceptance rate of masking sounds among the people exposed to such noises (Martin and Liebl, 2017). Previous works show that despite the success of masking sounds in increasing privacy and work efficiency in noisy environments, they come with the cost of increased annoyance and reduced working memory capacity of the participants (Poll et al., 2015). Hence, it is important to find ways to increase the efficiency of masking sounds by making them less annoying. In previous work, authors created a filter to create low-frequency dominance in the Speech-Shaped Noise as an alternative to conventional Speech-Shaped Noise (Pourfannan et al., 2023). The logic behind the proposed filter was the body of evidence suggesting that low-frequency ranges of the background noise contribute less to increasing the annoyance rate in the participants than the mid and high frequencies (Torija and Flindell, 2015). The effect is called “low-frequency dominance” and happens when there is a +30 dB difference between the low-frequency and mid and high-frequency ranges of the target noise. Hence, a filter was created to modify the SSN to amplify the low-frequency range and reduce the mid and high-frequency ranges while keeping the overall Long Term Average Spectrum (LTAS) of the noise similar to the speech signal. The obtained results showed that the annoyance rate for the generated SSN significantly reduced while the memory score of subjects was still significantly better than the no masking sound condition in the presence of background speech noise.

Despite the growing demand for the use of social robots in noisy public spaces (Steger, 2023), no previous study has investigated the effectiveness of using masking sounds to increase the efficiency of social robots implemented in noisy environments. Considering the large body of evidence indicating the negative effects of background noise in general, and speech signal more specifically, it is important to put efforts into developing principles for the combination of sound masking technologies into human-robot interaction designs for use in noisy environments. In an initial effort in this line of study, in the current experiment the effectiveness of using the low-pitch SSN as a masking sound was evaluated in a human-robot interaction in a noisy scenario, where the lecture given by the social robot was disturbed by irrelevant speech by several people in the background.

The hypothesis is that the performance of the subjects in the speech comprehension task, acoustic satisfaction, and sound privacy indexes will increase in the noisy condition with the low-pitch SSN as a masking sound in comparison with the same level of noise without using the proposed masking sound.

## 2 Methods

### 2.1 Participants

This study followed a within-subject design. Thirty-five subjects between the age of 18–30 years old (M = 21.9 SD = 0.6) participated in this experiment. Fourteen subjects were male, and twenty-one subjects were female. All participants were Japanese-speaking natives. To choose the appropriate sample size for the experiment an *a priori* power analysis was performed using G⋆ Power 3.1 (Faul et al., 2007) with the following input parameters: medium Effect size f = 0.25, *α* error probability = 0.05, and Power 1-*β* error probability = 0.90. The performed analysis showed that a sample size of around thirty-six subjects would be appropriate for this study. This research was reviewed and approved by the ethics committee for research involving human subjects at the Graduate School of Engineering Science, Osaka University.

### 2.2 Apparatus

A humanoid robot named CommU was used in this study. CommU is a versatile and portable on-table robot with an intuitive user interface which makes its implementation in different settings easy. The robot was positioned on an 80 × 60 cm desk and a chair was positioned for the participant in front of the robot at a distance of 100 cm. The height of the chair was measured to be at the same height as the robot and the robot’s gaze was fixed on the direction of the chair to create a sense of eye contact during the interaction. The robot was automatically set to blink every 15 s. The robot showed a total of seven expressive gestures during seven sentences out of the thirteen sentences of the lecture including nodding three times and raising a hand twice.

Two Jabra immersive 360-degree speakers with the model number “Speak 710–509 2018” were located on the two sides of the room, one beneath the speaking robot on the floor and the other one behind the participants at the same angle and distance from each other and the participant. The speakers supported the frequency band of 2402 MHz ∼2480 MHz. The speaker behind the participant played the conversation of three Japanese college students in a noisy bar about the result of their end-of-semester examinations during the whole experiment. The speaker beneath the robot played the low-pitch Speech-Shaped Noise during the masking sound condition and did not play anything in the no masking sound condition. The order of the conditions (masking sound *versus* no masking sound), and the order of stories (lecture 1 *versus* lecture 2) were counterbalanced so that each participant had an equal chance of experiencing each combination. To measure the ambient noise level in the room as well as the loudness of the robot’ voice, the intensity of the background noise, and the masking sound Mengshen’s digital sound pressure meter tool model M80A was used. The ambient noise in the room prior to conducting the experiment was measured at 30 dB A as a baseline. The loudness of the robot’s voice was set to 70 dB A. The loudness of the background noise ranged from a minimum of 45 dB A and a maximum of 57 dB A averaging at 50 dB A, and the masking sound was played with the constant loudness of 55 dB A.

### 2.3 Materials

Two short lectures were used in this experiment as speech material for the robot. The lectures were chosen to be about exotic fruits that most people do not have knowledge about to minimize the effect of prior information on the performance of the subjects. The lectures were translated from English to Japanese by an experienced Japanese translator and had the same length of 200 words in English and 450–500 characters in Japanese (see Supplementary Appendix A for the stories and related questionnaires). The task of the participants was to listen carefully to what the robot presented to them for a later quiz. After listening to each lecture, participants answered 7 multiple-choice questions about the content of the speech including main ideas and memory of the details. The final score of the subjects in the task was calculated as the sum of all their correct responses to the seven questions. The design of the experiment deliberately aimed to isolate the assessment of the sound channel in the encounter of the subject with the robot. Through the delivery of short robot lectures in a controlled setting, the effect of secondary factors such as the quality of interaction and participants’ experiences associated with the non-acoustic aspects of the social robot’s responses was minimized. Our main objective was to direct participants’ attention primarily to the sound channel and its evaluation, emphasizing this aspect over the overall quality or responses of the robot.

To measure the perceived understandability of the robot’s talk by the subjects, a questionnaire with four elements was used. The questions were answered on a 7-point Likert scale where 1 equaled strongly disagree, and 7 equaled strongly agree. The questions evaluated ease of understanding, ease of following the talk, ease of grasping the main ideas, and ease of remembering the key points of the talk. The questionnaire had an internal consistency of a = 0.96 (see Supplementary Appendix B for the questionnaire). Each question evaluated one aspect of the perceived experience of the subjects while listening to the lecture regarding their overall perceived understandability of the presented content. Since all four questions were shown to have a similar coefficient in the previous pilot studies, the final score of subjects for this index was the sum of their given score for all four questions without further conversion.

To measure the acoustic satisfaction and sound privacy of the subjects, the acoustic satisfaction Index established in previous research by (Veitch et al., 2002) was used. The words used in some of the questions of the index were modified to suit the experiment scenario used in this study. The index had two parts that measured the participants’ acoustic satisfaction and sound privacy separately. All questions were answered based on a 5-point Likert scale (see Supplementary Appendices C, D for the acoustic satisfaction and sound privacy indexes respectively). The acoustic satisfaction Index questions evaluated the perceived annoyance, loudness, interference between the voices, and the disturbances caused by noise in the participants. The sound privacy index started by asking the subject to imagine that they must work in this sound environment for hours. Then based on this imagination they were asked to answer five questions about their perceived distraction by others, interruption of work by others, ability to hold confidential meetings, the efficiency of speech, and ability to work for long hours.

The significance of sound privacy fluctuates based on the interaction context and speech content. Nevertheless, as sound privacy involves both minimizing distraction by reducing the overhearing of others’ conversations and ensuring one is not overheard, it has consistently served as a pivotal element in assessing the efficacy of different masking sounds in prior research. In this study, the score of subjects in both the acoustic satisfaction and the sound privacy index was the sum of their scores in that index without further conversion. Eventually, before finishing the experiment, subjects were asked to choose one of the two conditions they experienced (masking sound *versus* no masking sound) that they enjoyed more.

**FIGURE 1:**
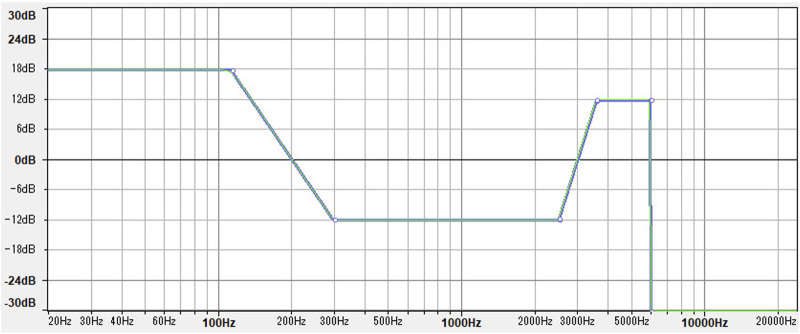
The filter used to create low-pitch Speech-Shaped Noise using Audacity.

To create the low-pitch Speech-Shaped Noise, the masking sound used in this experiment, the filter shown in Figure 1 was applied to the standard Speech-Shaped Noise generated by (Demonte, 2019) using the Audacity open source software. The filter basically consists of increasing the amplitude of the low-frequency band (16–125 Hz) by 18 dB A, while decreasing the amplitude of the mid and high-frequency bands (125–6,000) and cutting off all the frequencies above (6,000 Hz). Considering the importance of consonants for the intelligibility of speech signals (Fogerty and Humes, 2012), the frequency range responsible for the intelligibility of consonants (2,500–5,000 Hz) was increased in amplitude by 12 dB A to ensure a more powerful masking effect against the understandability of the intrusive speech signals. the resulting SSN was then normalized in loudness to the industry standard of −23 Loudness Units Full Scale (LUFS).

### 2.4 Procedure

**FIGURE 2:**
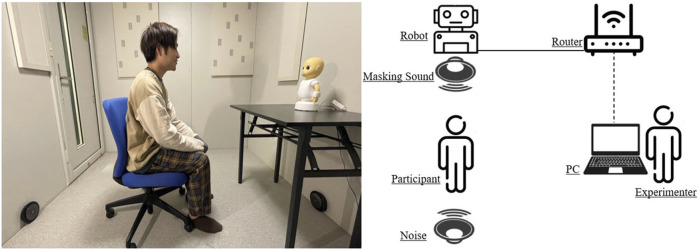
The setup used in the experiment.

**FIGURE 3:**
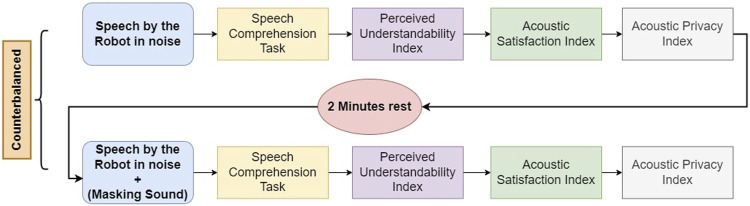
The flow of the conducted experiment for each subject.

The experiment was conducted in a 3 m × 2 m soundproof room in the Osaka University’s Engineering Sciences building. Participants filled out a written consent form before beginning the experiment and were paid a stipend after finishing the experiment. First, the procedure of the experiment was explained to the subjects. Then they were guided to the room where the experiment was conducted and was seated on a chair at a 1-m distance from the social robot. The robot was operated using an HTML connection by the experimenter. Figure 2 shows the setup used in this experiment. When each lecture by the robot finished, participants were guided outside the experiment room and answered the speech comprehension questionnaire followed by the perceived understandability, acoustic satisfaction, and sound privacy indexes. Each lecture took 2 minutes to finish. They could take a short break between the lectures upon their request. Figure 3 shows the experiment flow implemented in this study. At the end of the experiment, participants were asked to choose one of the conditions they experienced during the experiment based on how much they enjoyed it.

## 3 Results

### 3.1 Speech comprehension

**FIGURE 4:**
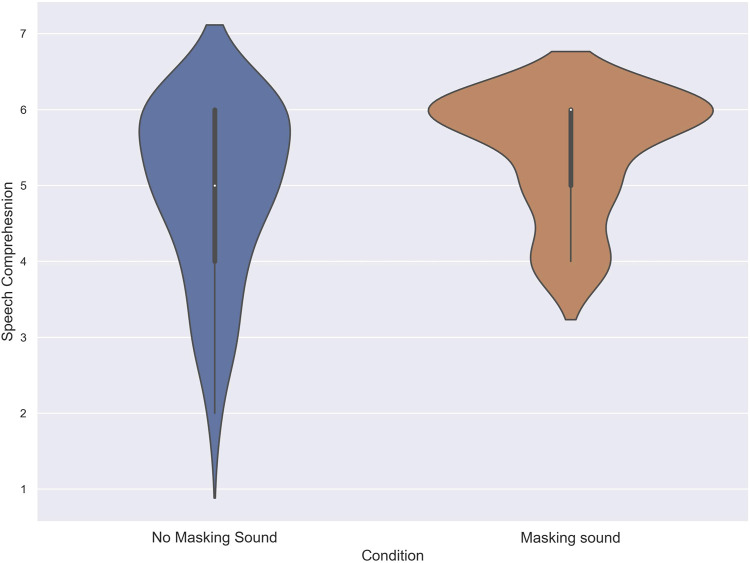
The score of subjects in the speech comprehension task in noise with and without using masking sound.

To assess the normality of the data distribution, Shapiro-Wilk tests were conducted on the acquired scores. The results indicated that the score of subjects in the speech comprehension task in both the no masking sound, and masking sound conditions deviated from the normal distribution (W = .82, *p* = .001), and (W = .67, *p* = .001) respectively. As a result, a Wilcoxon signed-rank test was conducted to compare the scores of subjects in each condition. The test revealed a statistically significant difference between the no masking sound, and masking sound conditions (T = 123, z = −4.38, *p* = 0.012, *δ* = .26). The median of the speech comprehension score for the masking sound was 6 compared to 5 for the no masking sound. Figure 4 illustrates the performance of subjects in the speech comprehension task for each condition.

### 3.2 Perceived understandability

**FIGURE 5:**
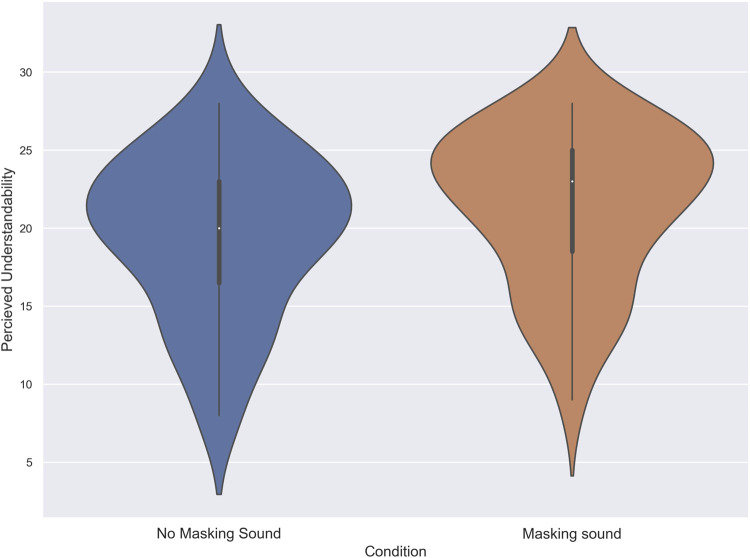
The score of subjects in the Perceived understandability task in noise with and without using masking sound.

To assess the normality of the data in the Perceived Understandability Index, the Shapiro-Wilk test was performed. The results of the Shapiro-Wilk test indicated that the data for the masking sound condition deviated from the normal distribution (W = .94, *p* = .01), while the data in the no masking sound condition followed a normal distribution (*p* = .11). Hence, a Wilcoxon signed-rank test was used to analyze the score of subjects in the perceived understandability index. The test revealed that the score of subjects in the masking sound condition (Mdn = 23) was significantly higher than the no masking sound condition (Mdn = 20) (T = 141, z = −2.85, *p* = 0.02, *δ* = .24). Figure 5 shows the evaluation of subjects in the Perceived understandability index for each condition.

### 3.3 Acoustic satisfaction

**FIGURE 6:**
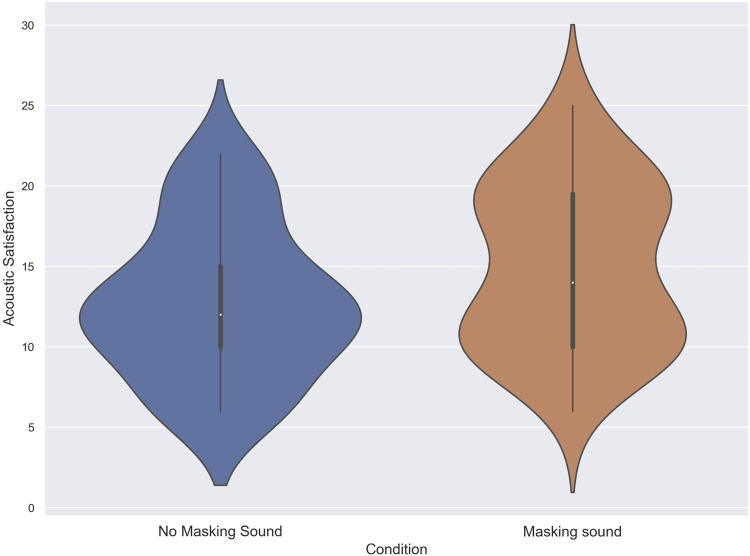
The score of subjects in the Acoustic satisfaction Index in noise with and without using masking sound.

The normality of the data for the acoustic satisfaction index was assessed using Shapiro-Wilk tests, revealing deviations from the normal distribution in both the no masking sound and masking sound conditions, with *p*-values of less than .01 (W = .94 and W = .93, respectively). Due to the non-normal distribution of the data, a Wilcoxon signed-rank test was performed to compare the scores of subjects in the no masking sound condition (Mdn = 12) with those in the masking sound condition (Mdn = 14) for the Acoustic Satisfaction Index. The results of the Wilcoxon test confirmed a significant difference in Acoustic satisfaction scores between the two conditions (T = 171, z = −2.36, *p* = 0.03, *δ* = .21) in favor of the masking sound. Figure 6 shows the score of subjects in the acoustic satisfaction index for each condition.

### 3.4 Sound privacy

**FIGURE 7:**
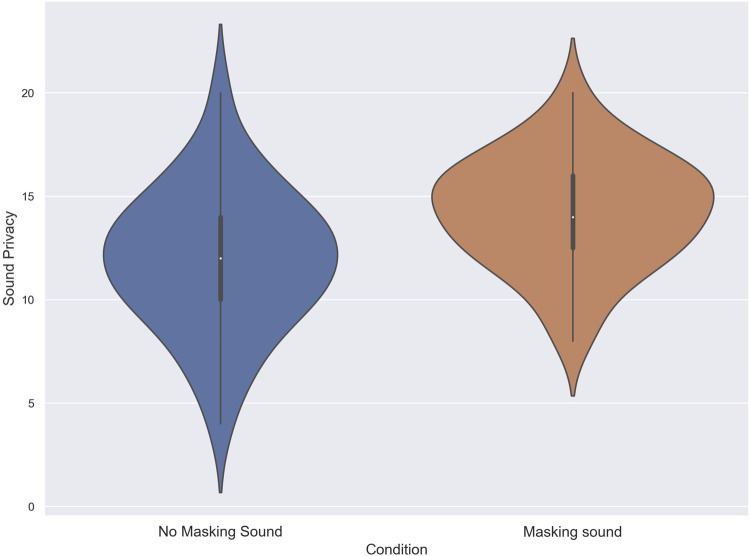
The score of subjects in the Sound privacy Index in noise with and without using masking sound.

Eventually, the results of the Shapiro-Wilk test indicated that the data for both the no masking sound and masking sound conditions conformed to a normal distribution (*p* = .76, and *p* = .42 respectively). Hence, a two-tailed dependent *t*-Test was used to analyze the scores of subjects in the Sound Privacy Index. The results from the two-tailed dependent *t*-Test revealed that the score of subjects in the masking sound condition (M = 14.26, SD = 2.69) was significantly higher than the no masking sound condition (M = 11.91, SD = 3.38), t (34) = 2.98, *p* < .00, d = .77. Figure 7 demonstrates the score of subjects in the Sound privacy index for each condition.

### 3.5 Preference of the subjects

**FIGURE 8:**
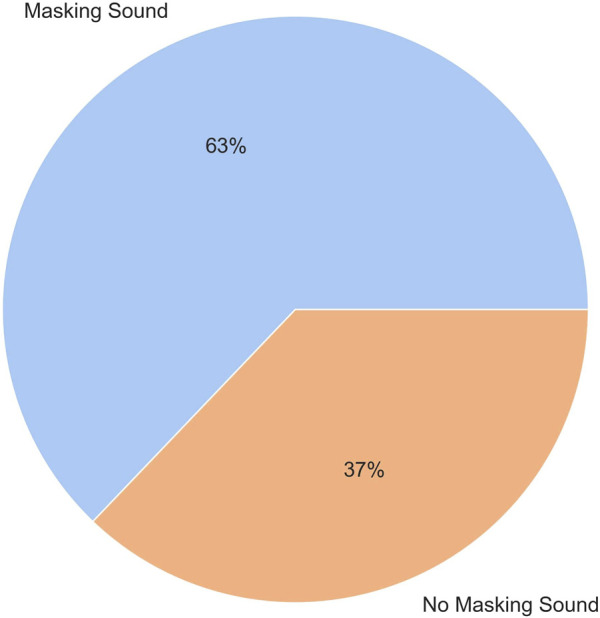
The preference of subjects for masking sound and no masking sound conditions in noise.

A Chi-Square Goodness of Fit Test was performed to determine whether the preference of participants for the robot’s speech was equal between the masking sound and no masking sound conditions. Out of the 35 subjects of the study, 22 subjects (63%) preferred the masking sound, while 13 subjects (37%) preferred the no masking sound condition. However, the proportion of preference did not differ significantly between the conditions |*X* ^2^| (1, 35) = 2.31, *p* = 0.25. Figure 8 shows the pie chart plot of the preference of subjects for each condition.

## 4 Discussion

The obtained result in the current study suggests that using a low-pitch Speech-Shaped Noise as the masking sound could significantly improve the comprehension of the robot’s speech by the participants as well as their subjective evaluation of their experience with the robot. In both the conditions of the current study, the robot gave a short lecture to the subjects with an intense level of noise present in the background which included both speech signals (three people talking in the same language as the subject) as well as ambient environmental noises that usually happen in a bar (babble noise, laughter, music, and people walking and moving chairs, *etc.*). The task of the participants was to listen carefully to the robot and ignore the presence of noise in the environment for a later quiz about the presented content. The robot’s monologue in this study diverged from the interactivity typically associated with conventional human-robot interaction experiments. The approach in this study involved delivering brief robot lectures on set topics in a controlled setting. This choice aimed to eliminate extraneous factors, including the subjective quality of interaction and participants’ experiences and impressions related to the social robot. The primary objective was to channel participants’ focus specifically on the acoustic aspect of the encounter with the robot and its evaluation, prioritizing this aspect over the overall quality or responses of the robot and their subtle effects on the impression of the subject.

Based on the obtained results, subjects could remember more information from the robot’s talk in the noisy environment when the lecture was presented concurrently with the masking sound in comparison with the same level of noise without adding the masking sound. The same pattern of the result was true for the components of the subjective evaluation of participants. Subjects rated their perceived understandability and acoustic satisfaction of the robot’s talk significantly higher when speech material was accompanied by the masking sound in noise. In addition, the highest positive effect was observed for the sound privacy index. Elements of the sound privacy index evaluated how much privacy subjects perceived while interacting with the social robot in the presence of other people’s speech in the near vicinity. Altogether, it can be inferred that the presence of the masking sound could indeed improve the cognitive performance of subjects in a relatively demanding task performed in a noisy social space, as well as their perceived quality of the acoustic encounter with the robot.

In general, Sound masking is a process of adding background stationary noise to an environment in order to mask or cover up unwanted noises in that environment (Bradley, 2003; Desai et al., 2012). The evolution of masking sounds has started from using randomly generated white noise to pink noise which takes into account the higher sensitivity of the human auditory system to higher frequencies to create a less annoying noise (Santhakumar, 2022), and eventually speech-shaped noise that mimics the spectral properties of the human speech signal into account to create more masking capability in comparison with previous masking sounds when the background noise includes human speech (Renz et al., 2018a). The limitation with the masking sounds, however, is that in general, they are not very advantageous in terms of acceptance rate among their users. This is due to the increased annoyance rate they cause, as well as an extra burden on the perceptual system of the users to ignore them (Martin and Liebl, 2017). The current work was built upon the previous advancements in the field and contributes to the literature by introducing the idea of adding low-frequency dominance to the speech-shaped noise as a potential remedy to reduce the previously observed annoyance in the users of the masking sound.

Previous research indicates that a higher proportion of low-frequency components compared to mid- and high-frequency ranges in the background noise, a phenomenon referred to as “low-frequency dominance,” is associated with reduced annoyance among individuals exposed to road traffic noise during the experiment (Torija and Flindell, 2015). This effect has been obtained in prior studies when the low-frequency components of the noise were amplified by at least 30 dB. This finding aligns with the key findings of Veitch et al. (Veitch et al., 2002), where they demonstrated that the same noise with a slope of −5 dB per octave was perceived as significantly less “hissy” when the low-frequency range (16, 31, and 63 Hz octave bands) was amplified, or, as the authors described it, “boosted.” Furthermore, when this modified noise was used as a masking sound, subjects reported improved speech intelligibility compared to the unmodified version of the same noise (Veitch et al., 2002). Hence, those researchers proposed a direct connection between acoustic satisfaction and the incorporation of low-frequency sounds in noise, and concluded that excessive high-frequency sounds should be minimized in masking sounds to enhance user performance. Other studies have also highlighted the positive impact of enhancing low frequencies in the masking sound on the acoustic satisfaction of individuals (Torija and Flindell, 2015). These collective findings have motivated the authors of this work to design a masking sound that follows the spectrum of human speech while being adjusted to minimize annoyance by incorporating a higher proportion of low frequencies to high frequencies in the noise.

As mentioned earlier, the results obtained in the current study also indicate that the proposed masking sound works effectively and could significantly increase acoustic satisfaction in the presence of intense background noise while making the concentration of participants on the information that the robot conveys easier. The findings of this study are important in regards that it is the first of its kind to investigate the effectiveness of using masking sounds concurrently with the robots talking in noisy social spaces to increase the efficiency of the human-robot interaction. This is of essential importance in crowded and noisy environments like airports and hospitals where robots are expected to convey vital information to human users, and it is important to convey the target information clearly to the subject to prevent any unwanted consequences.

It is noteworthy that in the current study, despite the significant increase in the score of subjects in the privacy index when using the proposed masking sound compared to the no masking sound condition, still, the score of subjects leans towards the lower end of the scale. Considering the type of noises used in this study (three individuals conversing in a noisy bar), lower scores in the no masking sound condition for the privacy index were expected. Although the addition of masking sound significantly improved this evaluation, the average score remained neutral, not exceeding the score of 3 on a 1-5 Likert scale. However, it is important to highlight that the Sound Privacy Index questions used in this study were originally designed for office and working environments, where a high level of privacy is crucial for tasks like long hours of work and confidential meetings. These design considerations, as outlined by (Veitch et al., 2002), explain the generally lower evaluation in the context of our experimental setup with background noise from a noisy bar that tried to mimic real-world public spaces. Hence, despite the lower scores, the substantial improvement to a neutral evaluation is still noteworthy. In future studies, in addition to designing specialized questionnaires to evaluate users’ acoustic satisfaction and perceived privacy tailored for Human-Robot Interaction scenarios in noisy environments, it is also important to run the experiments in real-world public spaces where such service robots are designed to serve society.

The current work has its own limitations that need to be considered in future studies. First of all, in the current study, the potential effect of the location of the loudspeakers is not taken into account. Conventionally, loudspeakers in a sound masking application are positioned in the ceilings. However, this might not be feasible in human-robot interaction scenarios in noisy public and social spaces where the location of the service robot might need to change due to specific demands of the environment. Previous works show that locating the loudspeakers in the ceiling is not very advantageous in comparison with the local positioning of the loudspeakers towards the direction of the intrusive noise (Renz et al., 2018b). In this study, we showed that a loudspeaker that is located just beneath the robot was still capable of creating the positive effect expected from the masking sound. However, in future studies, it is worth investigating to find out what is the most optimized location to implement the loudspeakers as well as whether it is possible to enhance this effect by designing a portable acoustic dome around the robots by implementing the loudspeakers and the sound absorbing partition screens in conjunction to create an even higher level of sound privacy and masking efficiency.

Another limitation of the current study is the reliance on questionnaires adapted from a comprehensive prior work on acoustic satisfaction in open-plan offices (Veitch et al., 2002) to evaluate the acoustic satisfaction and privacy of the subjects. While some modifications were made to the questions, the need for developing new indexes specifically tailored for assessing these fundamental concepts in the context of human-robot interaction in noisy environments is underscored. This consideration will be addressed in a future study. Another point to be noted about the current work is that in this study we did not use a visual display in conjunction with the robots. The augmentation of the presented auditory information by the robot using visual information is expected to be especially helpful in a noisy environment and is shown to enhance the memory of the subjects about the presented information (Heikkilä et al., 2017). The logic behind the decision not to include a visual display in this study was to exclude the effect of multimodality of the presented information and hence have a more controlled evaluation of the sole effect of adding masking sound on the performance of the subjects in the auditory domain before investigating the effect of stimuli from other modalities.

In this study, we aimed to determine if the proposed masking sound, the low-frequency dominant speech-shaped noise could enhance acoustic satisfaction, perceived sound privacy, and information retention in subjects listening to the robot’s speech in a noisy environment. To achieve this, we intentionally set up a controlled acoustic environment to evaluate the effectiveness of the proposed masking sound in comparison with the baseline with the same level of background noise but without any masking sound added to the environment. With significant results obtained in this experiment, the next step is to implement this setup in real-world situations with high levels of background noise. This step would be crucial to validate the current findings and evaluate its effectiveness in public spaces where the level and type of background noise cannot be controlled. Furthermore, in this study, we used the low-pitch Speech-Shaped Noise to keep the speech masking capabilities of the standard SSN while reducing its annoyance in the subjects. However, one might be interested in evaluating the effectiveness of the proposed masking sound in comparison with other types of masking sounds that use non-fluctuating natural sounds (e.g., rain sound and waterfall) and are designed specifically to increase acceptance rate among masking sound users (DeLoach et al., 2015) for performance comparison. The reason for using speech-shaped noise in this study is its similarity to speech signal which makes it a better candidate for blurring unwanted speech noise (Renz et al., 2018a) that is shown to be the most distracting type of background noise.

## 5 Conclusion

In this study, the effectiveness of a proposed masking sound which consisted of low-pitch Speech-Shaped Noise was evaluated to reduce distraction during a human-robot interaction in a noisy environment where the speech by the social robot was disturbed by several people talking in the background in a cocktail party type environment. Considering that social robots are increasingly being used in noisy public spaces such as airports, hospitals, and shopping malls, it is important to find solutions to compensate for the well-documented negative effect of noise on the cognitive performance of subjects as well as their user experience. No previous work has integrated sound masking technology into human-robot interaction designs. The results obtained in this study show that the proposed masking sound could reduce the distraction caused by background speech noise significantly. This is demonstrated in the significantly higher score of subjects in speech comprehension and perceived understandability tasks, as well as their evaluated acoustic satisfaction. Furthermore, subjects experienced a significantly higher level of sound privacy in the presence of the proposed masking sound.

The results obtained in this study provide a foundation for future works to investigate the remaining aspects of implementing sound masking technology into human-robot interaction settings. For instance, previous studies show that The location and positioning of the loudspeakers playing the masking sound have an impact on the effectiveness of the masking sound as well as the users’ perceived annoyance. Hence, it is worth investigating how this factor, i.e., the location of the source of the masking sound, could affect the quality of the human-robot interaction based on the position of the robot and its user in the target environment. Furthermore, previous studies suggest that factors such as height, material, and relative distance of the sound-absorbing partition screens when used in conjunction with the masking sound could increase the efficiency of sound masking in reducing distraction in open-plan offices. Hence, it is worth studying how this knowledge can be implemented to increase the quality of human-robot interaction in situations where more than one service robot is operated at the same time to provide information in noisy environments such as airports and train stations.

## Data Availability

The raw data supporting the conclusion of this article will be made available by the authors, without undue reservation.
