# Supplementary material for: Sound masking by a low-pitch speech-shaped noise improves a social robot’s talk in noisy environments
Source: Front Robot AI. 2024 Jan 9;10:1205209. doi: 10.3389/frobt.2023.1205209 (PMC10803579; doi:10.3389/frobt.2023.1205209)
Supplement: Supplementary file 1 [file DataSheet1.pdf]

A: MATERIAL FOR SPEECH COMPREHENSION TEST

A.1: Lecture 1 transcript

Recently I am learning about fruits and their nutritional value for humans. My favorite fruits are exotic ones that most people are not aware of. In this meeting, I want to talk about Mabolo. Mabolo is originally native to the Philippines, but it also grows in India and the Caribbean area. This fruit has a slightly unpleasant smell. People who have tried it would say its smell is more like cheese but as soon as you peel the fruit and refrigerate it for a few hours, the odor disappears. Some people like to season Mabolo with lemon juice and maple syrup. However, when mixed with syrups, the texture of the fruit turns a bit tough. So, it is better to fry it with butter and eat the crispy Mabolo with ham or spicy meat. The taste of the fruit is comparable to a mixture of apple and banana. That sounds yummy. Mabolo has a lot of medicinal uses. In ancient medicine, this fruit's leaves have been used to treat cough and fever. In addition, it is very rich in iron, fiber, and vitamin B. So, if you got a chance, you better try it once in your life at least.

A.1.1: Lecture 1 Questions

Table 1. Lecture 1 Questions

1	Which country was not mentioned as a place where Mabolo can grow?
2	What the smell of Mabolo is compared to?
3	How long Mabolo should be refrigerated so that its bad smell goes away?
4	What Mabolo is usually seasoned with?
5	What the smell of Mabolo is compared to?
6	Which of the below options is among the medicinal uses of Mabolo?
7	Which of the below options was not mentioned as a nutritional ingredient of Mabolo?

A.2: Lecture 2 transcript

Recently I am learning about fruits and their nutritional value for humans. My favorite fruits are exotic ones that most people are not aware of. In this meeting, I want to talk about Sugar Apple. Sugar apple is a pinecone-like fruit and is white inside when you peel it. The taste of sugar apple

assembles that of custard that’s why some people call it custard apple too. The edible portion coats the seeds generously, a bit like the gooey portion of a tomato. The seeds are scattered inside the fruit and are black and shiny, with a size of 12 millimeters each. But you should be careful as despite the tasty fruit itself, its seeds are poisonous and should be avoided. This fruit is also tropical and native to America and was introduced to Asia by Spanish merchandise. New variations of this fruit are being developed in Taiwan with even a better taste. The very sweet taste of sugar fruit is due to its high concentration of fructose inside it. Sugar apple is very rich in vitamin C, calcium, and riboflavin. The last one makes this fruit a good remedy for eye problems such as muscular degeneration and poor vision.

A.2.1: Lecture 2 Questions

Table 2. Lecture 2 Questions

1	What color is the inside of the sugar apple?
2	Which part of the sugar apple is edible?
3	Which vegetable is compared with sugar apple in the speech you heard?
4	How big sugar apple seeds can get?
5	Which nation introduced sugar apple to Asia?
6	Which country is developing a modified version of sugar apple?
7	Which ingredient of sugar apple makes it a good remedy for eye problems?

B: SUBJECTIVE EVALUATION INDEXES

B.1: The Perceived Understandability index

Table 3. The Perceived Understandability index

1	The lecture by the robot was easy
2	The lecture by the robot was understandable to me
3	I could easily understand what the lecture was about
4	I could easily understand what were the important points of the lecture

B.2: The Acoustic satisfaction Index

Table 4. The Acoustic satisfaction Index

1	Considering all the sounds I heard, I was satisfied with the sound environment
2	Please rate the loudness of the background speech heard during the experiment below
3	How much did the background speech you heard during the experiment interfere with hearing the robot's speech?
4	How would you rate the audio privacy (= not being heard by others in the next room) in this room?

B.3:The sound Privacy index

Table 5. The Acoustic Privacy Index

1	Even if I have a meeting in this environment, I think that the meeting can be held without being affected by other people's voices
2	I think I can work in this environment for long hours without being interrupted
3	The noise (background speech) in this environment was not distracting for me
4	I think that confidential conversations can easily be conducted in this environment
5	With this constant level of office noise, I think I can work efficiently
